# Impact of metabolic status on the incidence of psoriasis: a Korean nationwide cohort study

**DOI:** 10.1038/s41598-017-01983-y

**Published:** 2017-05-16

**Authors:** Eun Sook Kim, Kyungdo Han, Mee Kyoung Kim, Yong-Moon Park, Ki-Hyun Baek, Sung Dae Moon, Je-Ho Han, Ki-Ho Song, Hyuk-Sang Kwon

**Affiliations:** 1 Department of Internal Medicine, College of Medicine, The Catholic University of Korea, Seoul, 06591 Korea; 2 Department of Biostatistics, College of Medicine, The Catholic University of Korea, Seoul, 06591 Korea; 3 Epidemiology Branch, National Institute of Environmental Health Sciences, National Institutes of Health, Research Triangle Park, NC, 27709 USA; 4 Division of Endocrinology and Metabolism, Department of Internal Medicine, Incheon St. Mary’s hospital, Incheon, 21431 Korea; 5 Division of Endocrinology and Metabolism, Department of Internal Medicine, Yeouido St. Mary’s hospital, Seoul, 07345 Korea

## Abstract

Growing evidence suggests that obesity is a risk factor for incident psoriasis. This study was aimed to evaluate the association of obesity and metabolic status with the incidence of psoriasis. A total of 418,057 adults were followed-up using a nationwide prospective cohort study in Korea. Participants were stratified based on the body mass index categories and metabolic condition. During the follow-up visit, 11054 (2.6%) cases were found to have psoriasis. Diabetes, hypertension, hyperlipidemia, and obesity were all found to be risk factors for incident psoriasis. The metabolically unhealthy non-obese (MUNO) subjects (hazard ratio [HR], 1.29; 95% confidence interval [CI], 1.22–1.37) and metabolically unhealthy obese subjects (MUO; HR, 1.33; 95% CI, 1. 26–1.41) had a significantly higher risk of psoriasis incidence as compared to metabolically healthy non-obese subjects. The risk of psoriasis development was found to be high among the MUNO and MUO subjects in both sexes and all age groups. In conclusion, the metabolic health status was significantly associated with an increased risk of psoriasis in both obese and non-obese individuals. However, further studies are needed to evaluate whether the control of metabolic parameters can lower the incidence of psoriasis.

## Introduction

Psoriasis—a chronic disease characterized by patches of abnormal skin—is fairly common and affects 2–4% of the general population^1^. Psoriasis is an important health concern due to its adverse effects on physical, social, and psychological well-being, as well as the associated comorbidities. Approximately 30% of patients with psoriasis eventually develop psoriatic arthritis, which exhibits progressive worsening with a risk of joint damage^2^. Clinical data have indicated that psoriasis is associated with an increased prevalence of obesity, hypertension (HTN), hyperlipidemia, hyperglycemia, and smoking, all of which raise the risk of cardiovascular disease (CVD)^3, 4^. Moreover, a few population-based cohort studies recently showed an increased risk of incident myocardial infarction and stroke in patients with psoriasis. In fact, the risk appeared to be higher in a more severe form of psoriasis, suggesting that psoriasis could be a potential risk factor for CVD^5, 6^; however, the data are controversial^7, 8^.

Although the exact etiology remains unclear, psoriasis has been considered to be an immune-mediated inflammatory disease that is triggered by environmental factors in the background of genetic predisposition^9^. Epidemiologic studies have consistently indicated a relationship between psoriasis and obesity and metabolic disorders, and stated that obesity is a risk factor for psoriasis development and progression^10^. In contrast to obesity, metabolic disorders have been considered to represent the consequences of psoriasis, rather than as predisposing factors^3^. Given the close interrelationships among obesity, metabolic abnormalities, and psoriasis, it is expected that metabolic status would also affect the incidence of psoriasis. However, the data indicating such an association in a longitudinal manner are scarce.

In the present study, we aimed to investigate whether obesity and metabolic disorders may increase the risk of psoriasis in a nationwide prospective cohort.

## Materials and Methods

### Study population

In Korea, the National Health Insurance (NHI) compulsorily covers all Korean residents. In 2000, the National Health Insurance Service (NHIS) was launched under the supervision of the Ministry of Health and Welfare, and provides universal health coverage. The NHIS then established the nationwide National Sample Cohort (NHIS-NSC)—a database that provides public health information, such as the participants’ medical bill expenses claimed by medical service providers. A total of 1,025,340 nationally representative subjects were randomly selected from the Korean population in 2002, which amounts to ~2.2% of the population, and were followed until 2013^11^. Proportionate stratified random sampling was conducted based on a total of 1476 strata. The data of the cohort included insurance eligibility, medical care institutions, medical treatments, and health screening data. The NHIS provides general health checkups and a cancer screening program. All insured Koreans and their dependents can avail of free health checkups at ages >40 years, at least biannually, and individuals above a certain age can undergo examinations for specific cancers at 10% of the usual cost.

From this cohort, we selected subjects who had received health examinations at least once between 2002 and 2008. Among the 424,712 individuals in our follow-up study (2013), we excluded those aged <20 years. (n = 805) and those who were diagnosed with psoriasis prior to enrollment (n = 5,850). Thus, a total of 418,507 subjects were finally included. The patients in the study group who had developed psoriasis for the first time were identified based on the World Health Organization (WHO) International Classification of Diseases (ICD)-10 codes that represented psoriasis (L40*).

### Determinants of disease and demographic factors

The body mass index (BMI) was calculated by dividing the weight by the height squared (kg/m^2^), and was measured during regular medical check-up programs. Systolic and diastolic pressure was also measured at the time of weight measurement. Serum samples for measuring fasting glucose, hemoglobin, and total cholesterol levels were also obtained after an overnight fast at each examination site. Detailed histories of smoking status, alcohol consumption, and physical activity (including the amount and frequency) were obtained via a questionnaire. Statistical analyses were conducted using the simplified status classification of smoking (no, past, or current), alcohol (no, <2–3 times/month, ≥1 time/week), and physical activity (no activity, ≤4 times/week, or ≥5 times/week). The subjects’ socioeconomic status was categorized into 2 groups based on income level, which was dichotomized at lower 20% (<20% vs. ≥20%). The participants’ medical history was identified based on a combination of the following: clinic and pharmacy codes of ICD-10, list of prescribed medicine, and previous medical history. Obesity was defined based on a BMI of ≥25 kg/m^2^ and the metabolic unhealthy was defined as the presence of at least one of the following: DM, HTN, and hyperlipidemia. Subjects were divided into 4 groups based on the BMI and metabolic condition: metabolically healthy, non-obese (MHNO); metabolically unhealthy, non-obese (MUNO); metabolically healthy, obese (MHO); and metabolically unhealthy, obese (MUO).

### Statistical analysis

Statistical analyses were performed using SAS version 9.3 (SAS Institute, Cary, NC, USA). Continuous variables are expressed as mean ± standard deviation when normally distributed, or median (5–95% range) when the data are highly skewed. The continuous variables were compared using Student’s t-test, whereas the categorical variables were compared using the χ^2^ test. Variables with skewed distributions were assessed as continuous variables following log transformation.

The duration (person-years) of follow-up was determined from the enrollment date (2002–2008) to the date of psoriasis diagnosis, or the end of follow-up (December 2013), whichever was noted first. We conducted Cox proportional hazards analyses to estimate the hazard ratio (HR) and 95% confidence interval (CI) for the association of DM, HTN, hyperlipidemia, and BMI with incident psoriasis. The proportional hazards assumptions were evaluated by the logarithm of the cumulative hazards function based on the Kaplan-Meier estimates for each group. The overall disease-free rate was calculated using the Kaplan-Meier curve, whereas the log-rank test was used to examine the differences in the effect of the number of metabolic risk factors on psoriasis development. Moreover, we assessed the association between groups stratified according to obesity and metabolic status and incident psoriasis. Multivariable HRs were calculated after adjusting for age, sex, smoking, exercise, and income. Thereafter, we calculated the HRs of metabolic status for new-onset psoriasis, stratified by sex and age (20–39, 40–64, and >65 years). A *P* value of <0.05 was considered statistically significant.

## Results

**Figure 1:**
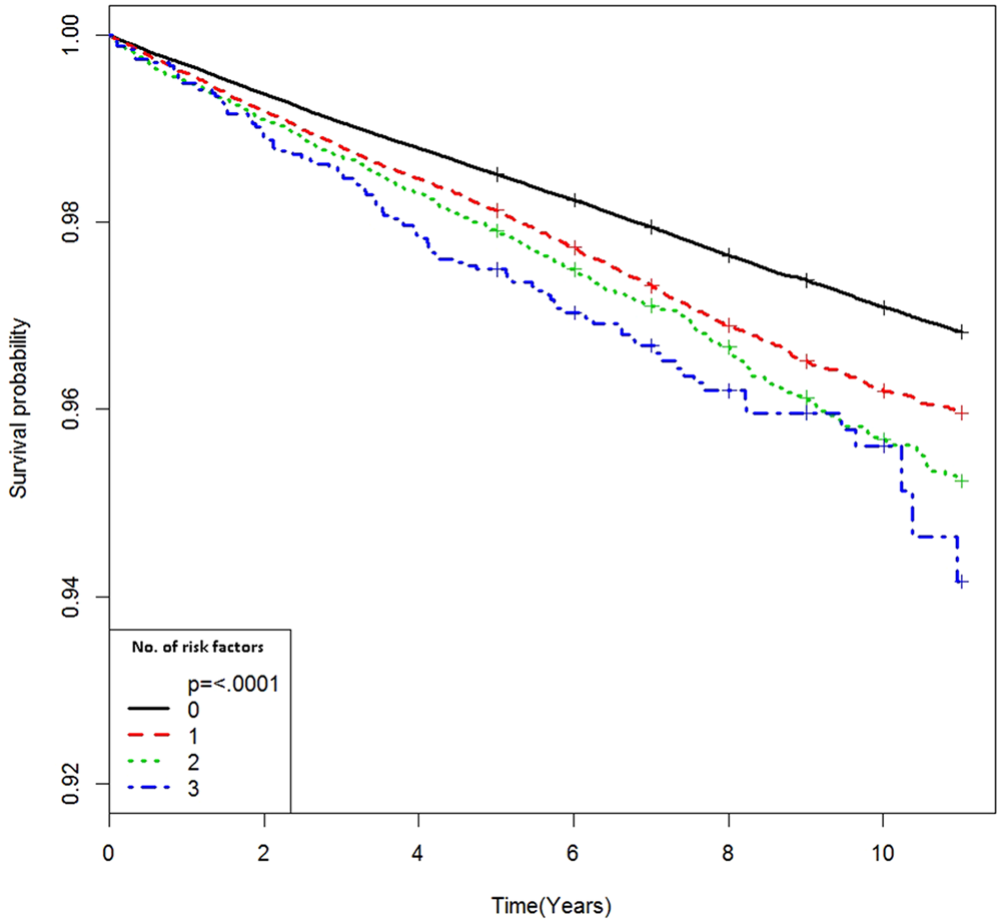
Kaplan-Meier estimates of survival curves for the time to incident psoriasis, stratified by metabolic risk factors. The median follow-up duration was 8.5 years. Subjects were divided into 4 groups according to the number of metabolic factors among diabetes mellitus, hypertension, and hyperlipidemia (log-rank test, P < 0.001).

Of a total of 418,057 participants included in the present study, 11,054 incident cases of psoriasis were identified over a mean follow-up duration of 8.5 years. As shown in Table 1, participants who developed incident psoriasis were more likely to be older, men, and smokers; have higher values of BMI, systolic blood pressure, diastolic blood pressure, fasting plasma glucose, and triglycerides; have higher alcohol consumption; and have a greater prevalence of diabetes mellitus (DM), HTN, and hyperlipidemia, as compared to those without psoriasis. Moreover, DM, HTN, hyperlipidemia, and obesity (BMI ≥ 25 kg/m^2^ vs. 18.5–22.9) were significant predictors of incident psoriasis (Table 2). The impact of the cumulative metabolic burden on psoriasis incidence was analyzed using Kaplan-Meier survival curves, based on the numbers of risk factors among DM, HTN, and hyperlipidemia (Fig. 1). Compared with subjects without any risk factors, those with more than 1 risk factor had a greater probability of developing psoriasis, in a dose-dependent manner (log-rank test, *P* < 0.001).

**Table 1:** Baseline characteristics according to the onset of psoriasis.

	New-onset psoriasis		*P*
	No (n = 407,003)	Yes (n = 11,054)	
Age (years, %)			<0.001
20–39	152,402 (37.4)	3,532 (32.0)	
40–64	210,449 (51.7)	6,074 (55.0)	
>65 years	44,152 (10.9)	1,448 (13.1)	
Male (%)	211,409 (51.9)	6,341 (57.4)	<0.001
BMI (kg/m^2^)	23.5 ± 3.2	23.7 ± 3.2	<0.001
SBP (mmHg)	123.5 ± 17.0	124.7 ± 17.3	<0.001
DBP (mmHg)	77.3 ± 11.2	77.9 ± 11.2	<0.001
FPG (mg/dL)	95.3 ± 28.4	96.4 ± 30.3	0.001
TC (mg/dL)	192.9 ± 37.8	194.4 ± 38.4	<0.001
AST (U/L)^a^	20.9 (20.9–20.9)	21.7 (21.5–21.9)	<0.001
ALT (U/L)^a^	23.3 (23.3–23.4)	23.9 (23.7–24.0)	<0.001
GGT (IU/L)^a^	24.4 (24.4–24.5)	25.7 (25.3–26.0)	<0.001
Smoking (%)			<0.001
No	252,844 (68.0)	6,406 (64.2)	<0.001
Past	15,444 (4.2)	451 (4.5)	
Current	103,562 (27.9)	3,116 (31.2)	
Alcohol (%)			0.046
No	202,692 (51.2)	5,439 (50.4)	
<2–3 times/month	77,936 (19.7)	2,092 (19.4)	
≥1 time/week	115,338 (29.1)	3,263 (30.2)	
Exercise			0.028
No	226,762 (57.2)	6,148 (57.1)	
≤4 times/week	138,239 (34.9)	3,699 (34.4)	
≥5 times/week	31,206 (7.9)	922 (8.6)	
Low income (%)	67,983 (16.7)	1,686 (15.3)	<0.001
DM (%)	16,376 (4.0)	555 (5.2)	<0.001
HTN (%)	47,732 (11.7)	1,622 (14.7)	<0.001
Hyperlipidemia (%)	16,845 (15.1)	570 (18.6)	<0.001
Metabolic status			<0.001
MHNO	185,730 (45.6)	4,159 (37.6)	
MUNO	97,779 (24.0)	3,286 (29.7)	
MHO	51,771 (12.7)	1,152 (10.4)	
MUO	71,723 (17.6)	2,457 (22.2)	

Data are expressed as mean ± standard deviation, median (95% confidence interval), or number percentage), unless otherwise indicated. BMI, body mass index; SBP, systolic blood pressure; DBP, diastolic blood pressure; FPG, fasting plasma glucose; TC, total cholesterol; AST, aspartate aminotransferase; ALT, alanine aminotransferase; GGT, gamma-glutamyl transferase; MHNO, metabolically healthy, non-obese; MUNO, metabolically unhealthy, non-obese; MHO, metabolically healthy, obese; MUO, metabolically unhealthy, obese; DM, diabetes mellitus; HTN, hypertension.

^a^Data were log-transformed prior to analysis.

**Table 2:** Longitudinal association between metabolic parameters and incident psoriasis.

	Cases	Person-years	Incidence rate^a^	HR (95% CI)		
				Unadjusted	Age-, Sex-adjusted	MV^a^-adjusted
DM	555	135,950	4.08	1.36 (1.25–1.48)	1.14 (1.04–1.25)	1.15 (1.05–1.26)
HTN	1,622	400,572	4.05	1.42 (1.33–1.50)	1.15 (1.08–1.23)	1.15 (1.08–1.24)
Hyperlipidemia	570	133,457	4.27	1.42 (1.31–1.55)	1.24 (1.13–1.35)	1.24 (1.13–1.36)
BMI (kg/m^2^)						
<18.5	416	160,815	2.59^a^	0.88 (0.79–0.97)	0.93 (0.84–1.03)	0.92 (0.83–1.03)
18.5–22.9	4,328	1,464,854	2.96	1	1	1
23–24.9	2,701	856,269	3.15	1.07 (1.02–1.12)	1.01 (0.96–1.06)	1.01 (0.96–1.06)
≥25	3,609	1,085,134	3.33	1.13 (1.08–1.18)	1.05 (1.01–1.10)	1.05 (1.00–1.10)
Metabolic status						
MHNO	4,159	1,605,863	2.6	1	1	1
MUNO	3,286	876,075	3.8	1.45 (1.38–1.52)	1.31 (1.24–1.38)	1.29 (1.22–1.37)
MHO	1,152	445,914	2.6	1.00 (0.93–1.07)	0.96 (0.89–1.02)	0.95 (0.89–1.02)
MUO	2,457	639,220	3.8	1.48 (1.41–1.56)	1.35 (1.28–1.42)	1.33 (1.26–1.41)

^a^per 1000 person-years. HR, hazard ratio; CI, confidence interval; DM, diabetes mellitus; HTN, hypertension; BMI, body mass index; MHNO, metabolically healthy, non-obese; MUNO, metabolically unhealthy, non-obese; MHO, metabolically healthy, obese; MUO, metabolically unhealthy, obese.

^a^MV includes age, sex, smoking, alcohol drinking, exercise, and income.

With regard to the metabolic status, the MUNO (HR, 1.29; 95% CI, 1.22–1.37) and MUO (HR, 1.33; 95% CI:1.26–1.41) subjects were associated with a significantly higher risk of psoriasis incidence as compared to the MHNO subjects, after adjusting for age, sex, smoking, exercise, and income. We also calculated the HRs of metabolic status for new-onset psoriasis according to sex and age (Table 3). Compared with the MHNO group, subjects from the MUNO and MUO groups had a higher risk of incident psoriasis among both men and women. Moreover, in the analysis stratified by age groups, all the subgroups exhibited significantly increased risks of psoriasis incidence; however, the HR was highest in the >65 years group, even though the difference was minimal.

**Table 3:** Association between metabolic status and incident psoriasis according to sex and age.

	Group	Incidence rate^a^	HR (95% CI)		
			Unadjusted	Age-, Sex-adjusted.	MV^b^-adjusted
**Sex**					
Male	MHNO	2.7	1	1	1
	MUNO	4.2	1.58 (1.49–1.68)	1.32 (1.24–1.42)	1.31 (1.22–1.41)
	MHO	2.7	1.00 (0.92–1.08)	1.01 (0.93–1.10)	1.03 (0.95–1.12)
	MUO	4.2	1.57 (1.47–1.68)	1.38 (1.29–1.48)	1.38 (1.28–1.49)
Female	MHNO	2.5	1	1	1
	MUNO	3.2	1.29 (1.21–1.39)	1.34 (1.23–1.45)	1.32 (1.22–1.44)
	MHO	2.4	0.95 (0.85–1.07	0.96 (0.86–1.08)	0.93 (0.82–1.05)
	MUO	3.4	1.36 (1.26–1.47)	1.41 (1.29–1.54)	1.39 (1.27–1.52)
**Age (years)**					
20–39	MHNO	2.5	1	1	1
	MUNO	3.4	1.39 (1.24–1.55)	1.40 (1.25–1.57)	1.34 (1.19–1.52)
	MHO	2.5	1.02 (0.93–1.12)	1.04 (0.94–1.14)	1.03 (0.93–1.14)
	MUO	3.3	1.31 (1.16–1.47)	1.33 (1.17–1.51)	1.34 (1.17–1.52)
40–64	MHNO	2.7	1	1	1
	MUNO	3.7	1.36 (1.27–1.44)	1.26 (1.18–1.345)	1.25 (1.17–1.34)
	MHO	2.6	0.96 (0.87–1.06)	0.93 (0.85–1.03)	0.94 (0.85–1.04)
	MUO	3.8	1.41 (1.32–1.51)	1.30 (1.21–1.39)	1.28 (1.19–1.38)
>65	MHNO	2.5	1	1	1
	MUNO	4.1	1.60 (1.34–1.90)	1.66 (1.40–1.98)	1.74 (1.44–2.11)
	MHO	2.8	1.08 (0.75–1.57)	1.14 (0.78–1.65)	1.24 (0.83–1.83)
	MUO	4.4	1.70 (1.42–2.05)	1.88 (1.56–2.27)	1.99 (1.62–2.44)

^a^per 1000 person-years.

^b^MV includes age, sex, smoking, alcohol drinking, exercise, and income.

HR, hazard ratio; CI, confidence interval; MHNO, metabolically healthy, non-obese; MUNO, metabolically unhealthy, non-obese; MHO, metabolically healthy, obese; MUO, metabolically unhealthy, obese.

## Discussion

In the present study, we observed that not only obesity, but also DM, HTN, and hyperlipidemia were all individually associated with an increasing risk of incident psoriasis. The results should be interpreted with caution considering that patients with psoriasis were identified by ICD-10 codes. Some individuals at the initial inclusion could have psoriasis but have not been formally given the diagnosis of psoriasis if they did not visit the hospital because of mild symptoms. However, these associations were found to be significant after adjusting for potential confounders, including age, sex, smoking, alcohol drinking, exercise, and income. In the analysis of the obesity subgroup, the metabolic status was found to be more important than obesity per se, as subjects with the MUNO subjects exhibited an increased risk of psoriasis development and those with the MHO did not. Similar trends were observed in both sexes and in all age groups.

Consistent with our findings, several previous studies have shown that obesity is associated with psoriasis; in fact, some studies have indicated that obesity may precede psoriasis development. A meta-analysis of 16 observational studies showed a graded association between psoriasis and BMI in a population of 2.1 million patients^12^. Moreover, prospective cohort studies have demonstrated that higher BMI is associated with incident psoriasis and weight gain^13, 14^, along with incident psoriatic arthritis^15^. Furthermore, a prospective randomized trial reported that diet-induced weight loss for 8 weeks reduced the disease severity in 60 overweight patients with psoriasis^16^. With regard to the metabolic disturbance observed in patients with psoriasis, previous studies have consistently shown a significantly positive association between psoriasis and metabolic disorders, and suggested that psoriasis may induce metabolic disorders via a proinflammatory milieu and subsequent insulin resistance^17^. Some data are available regarding the association between psoriasis and individual disease onset risk. Certain studies have reported that psoriasis is associated with a high risk of DM development^18–20^, but only few studies have assessed the association of psoriasis with HTN development^21, 22^. Nevertheless, most of the studies were cross-sectional in nature and hence lacked a temporal order. Moreover, to our knowledge, no data regarding the overall risk of metabolic disorders in cases of psoriasis have been published. The current data provide new insights that indicate metabolic disorders precede psoriasis, and that each disorder is a significant predictor of psoriasis incidence. This is in fact in contrast to the previous general assumption. Moreover, current evidence suggests that metabolic disorders have a potential role in the development of psoriasis, thus supporting our results. In the Nurses’ Health Study II, a prospective cohort study of 77,728 women in the US, Wu *et al*.^23^ reported that long-term hypertensive status is associated with an increased risk of psoriasis. In the same cohort, hyperlipidemia was also associated with an elevated risk of psoriasis and psoriatic arthritis^24^. In addition, a nationwide nested case-control study found that diabetic patients are at risk of first-time psoriasis^25^.

The mechanisms underlying the relationship between psoriasis and obesity and metabolic disorders remain unclear, although the shared immune-mediated pathogenesis may represent one explanation. Adipose tissue is composed of adipocytes and the stromal vascular fraction, including endothelial cells, preadipocytes, fibroblasts, and innate and adaptive immune cells, and exerts important functions at the crossroads of metabolism, immunity, and inflammation^26^. An overflow of saturated fatty acids in obesity induces insulin resistance and activates inflammatory cascades via toll-like receptors, endoplasmic reticulum stress, and inflammasome-mediated pathways^27^. Moreover, obesity induces a shift in the population of adipose tissue-resident immune cells, by increasing the number and function of proinflammatory cells (B-2 cells, M1-polarized macrophages, CD8^+^ T cells, and IFN-Υ^+^ Th1 cells) and inducing, a prototypic T helper 1 inflammatory response, while downregulating anti-inflammatory cells (regulatory T cells and Natural killer T cells)^27^. Thus, inflamed adipose tissue could promote low-grade systemic inflammation by releasing proinflammatory cytokines and chemokines (IL-1ß, TNF-α, IL-6, leptin, CCL2, CCL3, and CXCL8) and causing insulin resistance and endothelial dysfunction^28^. Given that the activation of Th1 and Th17 lymphocytes is a key mechanism of the keratinocyte-response pathway in psoriasis, and increased levels of circulating cytokines are observed along with the severity of psoriasis^29^, we believe that the inflammatory milieu induced by obesity could play a crucial role in triggering the initiation of psoriasis. Moreover, our data show that the obesity stratified by metabolic status was more closely associated with incident psoriasis as compared to obesity per se. This may be related to the increased amount of systemic inflammation observed within the same obesity range, as higher concentrations of complement C3, C-reactive protein, IFN, TNF-α, and IL-6, as well as lower levels of adiponectin, have been observed in the metabolic unhealthy group as compared to the metabolic healthy group^30, 31^. Second, insulin resistance may represent the link between the being metabolically obese and the pathogenesis of psoriasis, as increased insulin resistance is a key feature of obesity and metabolic disturbance^32^. Moreover, epidermal insulin resistance induced by proinflammatory cytokines has been reported to promote the persistent proliferation of keratinocytes^33^ and endothelial dysfunction, and contribute to the development of psoriatic plaques^34^. Third, the oxidative stress from metabolic disturbance could be responsible for the pathogenesis of psoriasis, based on the increased reactive oxygen species burden and decreased antioxidant capacity in various stages of psoriasis^35^.

Previous studies have speculated on the possibility of metabolic disturbance as a consequence of psoriasis, either as a result of the proinflammatory status of these patients, or as a result of unhealthy behaviors such as smoking, alcohol drinking, sedentary lifestyle, or bad eating habits, which are frequently observed among patients with psoriasis^3^. In contrast, our findings clearly suggest that obesity and metabolic disturbance precede psoriasis, and hence, they could be used as potential therapeutic targets to prevent psoriasis onset and progression. Psoriasis has a strong genetic component and approximately 40% of patients with psoriasis have family members with the disease^36^. Hence, the early detection and management of the metabolic unhealthy status of subjects with a family history of psoriasis may help prevent psoriasis both in the obese and non-obese population, in addition to the benefit of preventing CVD. Moreover, the treatment of metabolic disorders could be tailored in susceptible patients based on the effects of different types of drugs on psoriasis progression. For instance, a β-blocker—an anti-hypertensive drug—was found to mediate or aggravate psoriasis^23^, whereas short-term treatment with statins reduced the risk of psoriasis, although the long-term effects remain unclear^24^. With regard to anti-diabetic drugs, thiazolidinedione and Glucagon-like peptide-1 agonists were reported to have potential beneficial effects on psoriasis^37, 38^, whereas the use of insulin is associated with psoriasis development^25^. These clinical implications are more important in psoriasis due to its chronic and incurable nature.

The major strengths of the present study include the large sample size as well as the nationwide representative nature of the sample. Second, it was a prospective cohort study that ensured a thorough follow-up. In addition, the database used was stable, as it is maintained by the government or public institutions involved in providing national health information^39^. Third, the data contain demographic characteristics, including BMI, smoking, alcohol consumption, physical activity, and income status; hence, potential confounding factors could be suitably controlled for. Nevertheless, the current study has several limitations. First, information on psoriasis severity or psoriatic arthritis was unavailable, and hence, we could not evaluate the graded association of obesity and metabolic parameters with psoriasis. Second, no information was available on family history or drug use, which is a potential confounder of the association. Psoriasis is an immunologically mediated disorder that results from a complex interplay between genetic and environmental factors. It has been reported that risk of psoriasis is increased by three times in monozygotic twins compared to dizygotic twins. However, the concordance for psoriasis is as low as 35%, not 100%, which suggest that environmental factors also exert a vital role. Data on family history could determine the impact of metabolic parameters on psoriasis incidence independent of genetic influences^40^. Third, the use of diagnostic codes to infer psoriasis in claims may misclassify patients, even though the potential for such misclassification is minimal^41^. Moreover, there are possibility that initial population might have involved subjects with psoriasis, who did not receive clinical examination due to mild symptoms. Fourth, the present study included only Korean subjects, and hence, it is unclear whether these findings can be generalized to other ethnicities. Fifth, some residual confounding as a result of unmeasured factors cannot be excluded.

In conclusion, the metabolic health status was significantly associated with an increased risk of regardless obesity in this nationwide representative, prospective cohort. However, these associations should be interpreted cautiously due to the limitation of the claims-based diagnosis of psoriasis. Further studies are needed to evaluate whether the control of metabolic parameters can lower the incidence of psoriasis.
